# Diagnostic workup of endocrine dysfunction in recurrent pregnancy loss: a cross-sectional study in Northeast China

**DOI:** 10.3389/fendo.2023.1215469

**Published:** 2023-09-18

**Authors:** Liyang Zhang, Yushu Du, Jingshuang Zhou, Jiapo Li, Hongfei Shen, Yilin Liu, Chuanyang Liu, Chong Qiao

**Affiliations:** Obstetrics and Gynaecology Department, Shengjing Hospital of China Medical University, Shenyang, China

**Keywords:** recurrent miscarriage, PCOS, hyperprolactinemia, obesity, vitamin D, thyroid dysfunction, blood glucose abnormality

## Abstract

**Objective:**

To evaluate the prevalence of abnormal endocrine dysfunction for recurrent pregnancy loss (RPL) amongst patients with two versus three or more pregnancy losses.

**Methods:**

This cross-sectional study retrospectively collected pre-pregnancy data of 537 women diagnosed with RPL in Shengjing Hospital of China Medical University from 2017 to 2022, including the baseline data of patients and the test results of endocrine factors. Several endocrine dysfunction included in this study were: thyroid dysfunction, obesity, hyperprolactinemia, polycystic ovary syndrome and blood glucose abnormality. Furthermore, vitamin D level were collected to study its relationship with endocrine dysfunction. Finally, we subdivided the patients according to the number of previous pregnancy loss and compared the prevalence of endocrine dysfunction between subgroups.

**Results:**

Among 537 RPL patients, 278 (51.8%) patients had abnormal endocrine test results. The highest incidence of endocrine dysfunction was thyroid dysfunction (24.39%, 131/537), followed by hyperprolactinemia (17.34%, 85/490), obesity (10.8%, 58/537), polycystic ovary syndrome (10.50%, 56/533), and abnormal blood glucose (5.29%, 27/510). Only 2.47%(13/527) of patients have vitamin D level that reach the standard. After subdividing the population according to the number of pregnancy loss, we did not find that the incidence of endocrine dysfunction (P=0.813), thyroid dysfunction (P=0.905), hyperprolactinemia (P=0.265), polycystic ovary syndrome (P=0.638), blood glucose abnormality (P=0.616) and vitamin D deficiency (P=0.908) were different among patients with two versus three or more pregnancy losses. However, obesity (P=0.003) was found more frequently observed in patients with more times of pregnancy loss.

**Conclusion:**

The prevalence of endocrine dysfunction in RPL population is high. There is no difference in the prevalence of endocrine dysfunction, except for obesity, among patients with two or more pregnancy losses, which may suggest investigations of endocrine dysfunction when patients have two pregnancy losses.

## Introduction

Recurrent miscarriage can be devastating for women who wish to have children, with a global prevalence ranging from 1 to 3% (1). Individuals who experience recurrent miscarriage are at increased risk of many obstetric complications, as are the emotional and psychological harms of miscarriage. This is not the end of the physical and psychological consequences of recurrent miscarriages, since complex etiologic screening and expensive systemic therapy can also be financially stressful for a family (2). Endocrinological factors are now a critical component of the screening process used by clinicians to screen patients for recurrent miscarriage. The secretion of hormones such as thyroid stimulating hormone and prolactin has an irreplaceable impact on pregnancy outcomes. Such as prolactin, it may play an important role in maintaining corpus luteum function and progesterone secretion, with potential impact on the establishment of pregnancy (3). Hyperprolactinaemia plays a critical role in infertility because it suppresses the production of GnRH and thus pituitary gonadotropins (4). The most common endocrine abnormalities seen in patients with recurrent miscarriage are luteal phase defect, polycystic ovary syndrome, thyroid dysfunction, obesity, and hyperprolactinaemia. The incidence and risks of these endocrine abnormalities in recurrent miscarriage have been the subject of many independent studies, but studies systematically describing the proportion of endocrine factor abnormalities in patients with recurrent miscarriage are still lacking.

Not only are screening factors varied and complex, but the timing of screening is also a challenge in current research on recurrent miscarriage (5). Due to regional differences in the definition of recurrent miscarriage and clinicians’ own practice experience, some have begun routine etiologic screening for recurrent miscarriage following two pregnancy losses, whereas others wait for a third or more pregnancy losses before initiating etiologic screening. Based on these two points, the aims of this study include (1): To describe the proportion of endocrine dysfunction in patients with recurrent miscarriages (2). To compare whether there are differences in endocrine dysfunction between patients with recurrent miscarriages with different numbers of miscarriages.

## Materials and methods

### Study sample

The China Medical University Birth Cohort is an ongoing prospective cohort study that includes a sub-cohort of patients with recurrent miscarriage specifically enrolled in the Recurrent Miscarriage Clinic. In this cross-sectional study, data from 2017 to 2022 are collected from the recurrent miscarriage clinic at Shengjing Hospital, a local tertiary center, with patients being enrolled according to the following criteria: 1) Patients with two or more pregnancy losses and have completed a detailed history taking form of pregnancy losses. 2)Patients who have received a comprehensive aetiological screening prior to conception for recurrent miscarriage regarding endocrine factors (including thyroid dysfunction, obesity, hyperprolactinemia, polycystic ovary syndrome and blood glucose abnormality) at our hospital. 3) Patients did not take medicines that may affect the test results (including traditional Chinese medicine) before screening. Whereas patients with abnormal results of the endocrine factors on the initial examination will need at least a second repeat examination to make the diagnosis. Simple random sampling was adopted for the study.

Pregnancy loss as defined in this study was urine/blood β-hCG positive or ultrasound-confirmed pregnancy sacs. Pregnancy loss was defined as any spontaneous pregnancy loss or fetal weight ≤ 500g before 20 weeks. Molar pregnancy, ectopic pregnancy, implantation failure and pregnancy terminations were excluded from the analysis.

### Sample-size calculation

The sample size was calculated using an online sample calculation website, http://riskcalc.org:3838/samplesize/. The collection of data from 100 cases in the preliminary stage allowed us to roughly determine the proportion of various endocrine abnormalities in the three populations (patients with two/three/four and more pregnancy losses). Glucose abnormalities were the type of endocrine abnormality that had the lowest prevalence and had a prevalence of approximately 5%. The Type I error rate set in this study was 0.05, the degree of certainty of the study was 0.8, and the approximate ratio of the sample size between the three populations was 1. A minimum sample size of 480 was calculated for this study.

### Data collecting

A patient history collection form is used to collect baseline characteristics, which asks the patient to describe in detail her menstrual history and maternal history, including the number of miscarriages, the cause of the miscarriages, and the presence of ultrasound images to determine the occurrence of early intrauterine pregnancy. The clinician reviews this information (usually with more detailed questioning) in order to clarify the accuracy of the data collected. Whereas outcome-related data are collected primarily through the collection of laboratory test results obtained from the hospital’s electronic medical record system. We do not capture data from tests conducted by patients at other hospitals, since differences in the kits can lead to inaccurate results.

The baseline characteristics collected were: age, height, weight, number of pregnancies and number of pregnancy losses. Outcome data collected included PCOS, thyroid dysfunction, hyperprolactinaemia, blood glucose abnormality, obesity, and levels of vitamin D. Premature ovarian failure was removed from the outcome events because of a small number of cases; luteal phase defect was removed from the outcome events because of diagnostic challenges. All baseline data were collected from the patients on their first visit to the recurrent miscarriage clinic. Data on all disease diagnoses were obtained in the non-pregnant state.

### Diagnostic criteria

The diagnosis of PCOS is based on the revised Rotterdam diagnosis (6). Thyroid dysfunction is categorized as abnormal thyroid autoimmune antibodies alone, elevated or reduced TSH levels alone, and abnormal TSH levels in combination with abnormal levels of autoimmune antibodies; An abnormal TSH is diagnosed as a TSH of less than 0.3 μIU/ml or more than 4.8 μIU/ml (Specific reference values established for non-pregnant local normal individuals). Hyperprolactinaemia was defined as PRL greater than 26.72 ng/ml. abnormal blood glucose was classified as impaired glucose tolerance (6.1 mmol/L ≤ fasting glucose <7.0 mmol/L), and diabetes mellitus (fasting glucose ≥7.0 mmol/L). Diagnosis of obesity is based on the criteria for diagnosis of obesity developed by the working group on obesity in China (7). Endocrine dysfunction is determined by a combination of PCOS, abnormalities in thyroid function, hyperprolactinaemia, glucose abnormalities, and obesity; if any of these abnormalities are present, the patient is considered to have an endocrine dysfunction. Furthermore, because vitamin D may affect many endocrine factors in patients with recurrent miscarriages, vitamin D levels were also included in the present study and examined as a separate outcome; the concentration of Vitamin D was measured as 25 hydroxyvitamin D. 25-OH Vit D ≤ 20ng/ml was diagnosed as a vitamin D deficiency; A diagnosis of vitamin D insufficiency was made if 20 < 25-OH Vit D ≤ 30ng/ml. Patients were asked to undergo tests related to recurrent pregnancy loss when they are nonpregnant and at least three months after their last pregnancy loss. Patients undergoing these laboratory tests are advised to avoid cold or menstrual periods, which can affect the results of laboratory tests. Screening time for hormones is 1-3 days of menstruation.

### Analyzed data

For comparisons of baseline data, the information about the measure is expressed as the mean ± the standard deviation. Counts are shown as quartiles. Comparisons of endocrine abnormality rates between those with two and three miscarriages, and those with more than three miscarriages, were made using a two-sided Pearson chi-square test. Since multiple group comparisons were performed, we used Bonferroni correction. The vitamin D comparisons were analyzed by ANOVA. All data analysis was carried out in SPSS, windows, version 25.

## Results

### General situation of patients and the proportion of endocrine dysfunction

The total number of RPL patients included in this study was 537, including 278 patients with a diagnosis of endocrine dysfunction, comprising 51.8% of RPL patients. Fifty-six of these patients were diagnosed with PCOS (10.50%, 56/533); 85 patients were diagnosed with hyperprolactinaemia (17.34%, 85/490); Twenty-seven patients had a diagnosis of blood glucose abnormality (5.29%, 27/510); 58 patients were diagnosed with obesity (10.80%, 58/537). A total of 537 patients had their vitamin D levels tested, and the average vitamin D value was 15.11 ± 6.42ng/ml. 83.49% of these patients, (440/527) had a diagnosis of vitamin D deficiency, 14.04% (74/537) were diagnosed with vitamin D insufficiency, and only 2.47% (13/527) of patients had vitamin D levels that were up to the standard. A total of 131 patients (24.39%) were diagnosed with thyroid function abnormalities (131/537). 101 of the patients with abnormal thyroid function were found to have abnormal autoantibodies alone, representing 77.1% of patients with abnormal thyroid function (101/131); Eighteen had an abnormal TSH level alone, representing 13.74% of patients with an abnormal thyroid function (13/131); and 12 had combined autoantibodies and TSH levels, accounting for 9.16% of patients with abnormal thyroid. Among 113 patients with positive autoimmune thyroid antibodies, 39.82% (45/113) were found to be positive for anti-Tg alone; Of these, 17.70% (20/113) were positive for anti-Tpo antibody alone; and 42.48% (48/113) were positive for both antibodies. Further details can be found in **Tables 1** and **2**.

**Table 1:** Baseline characteristics and the proportion of endocrine disorders.

Characteristic	n(%)
Age	
20-29	133(24.77)
30-34	272(51.03)
35-45	132(24.58)
BMI	
<18.5	29(5.40%)
18.5-23.9	321(59.78%)
24-28	129(24.02%)
>28 (obesity)	58(10.8%)
Times of pregnancy loss	
2	297(55.31)
3	184(34.52)
More than 3	56(10.51)
PCOS	
No	477(89.50)
Yes	56(10.50)
Thyroid Dysfunction	
No	406(75.61)
Yes	131(24.39)
HPRL	
No	405(82.66)
Yes	85(17.34)
Blood glucose abnormality	
No	483(94.71)
IGT	23(4.51)
DM	4(0.78)
Vitamin D level	
Normal	13(2.47)
Insufficient	74(14.04)
Deficiency	440(83.49)

**Table 2:** Comparison of endocrine disorders among patients with different numbers of pregnancy loss.

	Times of pregnancy loss			P value
	2	3	≥4	
Endocrine dysfunction				0.817
No	146(56.4%)	88(34.0%)	25(9.7%)	
Yes	151(54.3%)	96(34.5%)	31(11.2%)	
PCOS				0.638
No	261(54.7%)	166(34.8%)	50(10.5%)	
Yes	34(60.7%)	16(28.6%)	6(10.7%)	
Thyroid Dysfunction				0.905
No	225(55.4%)	140(34.5%)	41(10.1%)	
Yes	72(55.0%)	44(33.6%)	15(11.5%)	
HPRL				0.265
No	220(53.9%)	143(35.0%)	45(11.0%)	
Yes	54(63.5%)	24(28.2%)	7(8.2%)	
Blood glucose abnormality				0.616
No	272(56.3%)	161(33.3%)	50(10.4%)	
IGT	10(43.5%)	9(39.1%)	4(17.4%)	
DM	2(50.0%)	2(50.0%)	0(0.0%)	
Vitamin D level				0.908
Normal	7(53.8%)	4(30.8%)	2(15.4%)	
Insufficient	38(51.4%)	27(36.5%)	9(12.2%)	
Deficiency	248(56.4%)	147(33.4%)	45(10.2%)	
Obesity				0.003*
No	277(57.8%)	155(32.4%)	47(9.8%)	
Yes	20(34.5%)	29(50.0%)	9(15.5%)	

* means P value<0.05.

### Multiple endocrine dysfunctions

When analyzing whether patients presented with a combination of multiple endocrine disorders, we found that 74.46% (207/278) of patients with RPL had only one endocrine disorder;

23.02% (64/278) had a combination of two endocrine disorders; 2.15% (6/278) had a combination of three endocrine disorders, and only 1 patient had a combination of 4 endocrine disorders. To further examine whether there was an interaction between the various endocrine disorders, or what types of endocrine disorders were seen more frequently together, the association between each of the endocrine factors was analyzed. Although we did not find a significant association between any two endocrine disorders (results not shown), there was a tendency for PCOS to be associated with obesity (p=0.059), which is consistent with our clinical knowledge.

### Endocrine dysfunction and the number of pregnancy losses

Comparisons of subgroups revealed that PCOS, thyroid dysfunction, hyperprolactinaemia, blood glucose abnormality, and vitamin D levels were not significantly different between groups. There was a significant difference in obesity between patients with different numbers of pregnancy losses (p=0.003). We then stratified the patients by age for different numbers of pregnancy losses and showed that all endocrine disorders were not associated with the number of miscarriages in the subgroups less than 30 years and greater than or equal to 35 years. On the other hand, among those above or equal to age 30 and below age 35, obesity was the only factor that was significantly different between patients with different numbers of pregnancy losses (p=0.017).

## Discussion

### Main findings

This cross-sectional study describes the proportion of endocrine disorders in the recurrent miscarriage population and compares whether there are differences in the distribution of endocrine disorders among those with two, three, and more pregnancy losses. Except for obesity, our results did not find a significant association between the number of pregnancy losses and the distribution of endocrine disorders. This finding may suggest that clinicians need to begin screening for endocrine factors at the beginning of two miscarriages in order to intervene early and prevent patients from experiencing further miscarriages.

### Thyroid dysfunction

Currently, abnormal thyroid function is a hot topic in recurrent miscarriage research. Abnormalities in thyroid function typically include both abnormal TSH levels and abnormal autoimmune antibodies. Many clinical studies have been conducted to examine whether abnormal thyroid function can lead to pregnancy loss and other adverse pregnancy outcomes. Recent research suggests that abnormal levels of TSH alone or the presence of thyroid antibodies are associated with pregnancy loss (8). In a meta-analysis of 22 studies, high serum thyroid antibody levels have been shown to lead to recurrent miscarriage and the use of T4 replacement therapy is beneficial in pregnant patients with recurrent miscarriage (9). However, a randomized clinical trial carried in 2019 concluded that that the use of levothyroxine in euthyroid women with thyroid peroxidase antibodies did not result in a higher rate of live births than placebo in normal population (10), and the preconception use of levothyroxine in recurrent miscarriage population still need further research. The mechanism by which thyroid antibodies contribute to poor pregnancy outcomes may be linked to their action on immune cells in the endothelium; studies have shown that the secretion of IL-4 and IL-10 is significantly reduced in endothelial T cells and that the expression of interferon-γ is significantly increased in antibody- positive patients (11). Likewise, polyclonal B cells were overexpressed in patients with autoimmune thyroid disease, and toxic NK cell migration was significantly enhanced (12). There were no significant differences in the prevalence of thyroid dysfunction according to the number of pregnancy losses reported in this paper. These findings are also similar to previous studies.

### PCOS

The second endocrine abnormality that is the focus of this paper is PCOS, whose prevalence in the recurrent miscarriage population remains a mystery. The reported incidence of polycystic ovarian changes on ultrasound imaging in the RPL population has been reported to range from 4.8 to 82% (13–16). In a meta-analysis published in 2016, the authors included 15 articles that used the Rotterdam diagnosis as a diagnostic criterion for PCOS and concluded that the average prevalence of PCOS in the general population was 10% (17). In the RPL population, the prevalence of PCOS were reported to be 14.3% (18). Common symptoms of PCOS include insulin resistance and hyperinsulinaemia, both of which are independent risk factors for pregnancy loss (13, 19, 20). Therefore, it is important to intervene before pregnancy for PCOS patients to improve miscarriage rates. Unfortunately, there is still a lack of research on how to manage during pregnancy in PCOS patients. Researchers have found that metformin treatment might reduce the risk of miscarriage in PCOS patients (21). However, more research is needed on the safety of medication and its use in the population with recurrent miscarriage.

### Obesity and glucose abnormality

Of note, about 35% of PCOS patients have a combination of obesity, which is believed to be associated with pregnancy loss (22, 23). The prevalence of obesity has been increasing in various countries in recent decades (24). The prevalence of obesity in women of reproductive age (20-39 years) in the United States rose from 28.4% to 34% between 1999 and 2008 (25). In China, the prevalence of obesity in adults rose from 3.6% in 1992 to 14.0% in 2014 (26). The hypothalamic-pituitary-ovarian axis is disrupted by obesity, and overweight women have a shorter luteal phase and lower levels of follicle-stimulating hormone, luteinizing hormone, and progesterone (27). However, in the current study, we found that obese patients were more common among women with a greater number of pregnancy losses. Obesity remained associated with the number of pregnancy losses among those aged thirty to thirty-four years even after stratifying for age. It is noteworthy that the subgroup sample sizes of the study population did not achieve the minimum sample size considered for validity following patient stratification, therefore, a larger sample size is required to confirm the association of obesity with pregnancy loss. As with obesity, blood glucose abnormality is also highly associated with pregnancy loss. In a recently published cross-sectional study, the authors suggest that women with recurrent miscarriages are more likely to have impaired β-cell function and abnormal glucose metabolism (28). Hyperglycaemia can inhibit the differentiation of trophoblasts and thereby interfere with implantation, increase oxidative stress, and affect the expression of key genes that are essential for embryogenesis (29). Hyperglycaemia promotes pregnancy loss through the promotion of premature programmed cell death of key progenitor cells within blastocysts (30). In this study, 5.29% of patients had abnormal blood glucose levels. Whereas the prevalence of diabetes among Chinese women of all ages was as high as 11%, the lower prevalence in this study may be due to the occurrence of diabetes being more prevalent in the elderly and in more economically developed regions.

### Hyperprolatinaemia

Prolactin is a hormone secreted from the lactotrophic cells in the anterior pituitary. In a randomized trial, Hirahara et al. found that high levels of prolactin increased the risk of pregnancy loss in women with RPL (31). A cross-sectional study of 69 women with RPL and 31 women of reproductive age and 30 women with infertility found that the prevalence of hyperprolactinaemia was similar across groups, though it was highest in the infertility arm and not in the RPL group (32). While the deleterious effects of hyperprolactinaemia in the recurrent miscarriage population remain unclear, due to its potential risk of producing infertility and the patient’s desire for children, pre-pregnancy medication is still needed.

### Vitamin D

Lastly, the article also analyzed the incidence of vitamin D levels in individuals with different numbers of pregnancy losses and found no significant differences. Vitamin D concentrations are now considered essential for maintaining pregnancy. Endometrium with a greater number of vitamin D receptors is more likely to conceive, while vitamin D deficiency is more likely to result in miscarriage (33). Vitamin D deficiency can result in the development of numerous endocrine defects including PCOS, autoimmune thyroid disease, diabetes, and obesity (34). Despite such an important role of vitamin D in pregnancy, vitamin D insufficiency or deficiency is currently prevalent in the population. In this study, only 2.47% of patients had a normal vitamin D level, whereas 83.49% were diagnosed with a vitamin D deficiency level. Consistent with our results, Li et al. found that as many as 70% of women during pregnancy were deficient in vitamin D levels, and only 1.6% reached normal levels (35). Though the study population is different, the results of our study and those of Li et al. may suggest the high prevalence of insufficient and deficient vitamin D levels in both the pregnant and non-pregnant women in China. This study also suggests that vitamin D insufficiency and deficiency are not associated with the number of pregnancy losses in patients with recurrent miscarriage. Additional studies are needed to determine if vitamin D supplementation can ameliorate miscarriage.

### Bias

Bias in cross-sectional studies includes many aspects and a variety of classifications of bias are summarised in the study by Wang et al (36). Because only baseline data collection involved question-based or questionnaire-based data collection (and this data was reviewed by clinicians), non-response bias, loss-to-follow-up bias, observer bias, interviewer bias, and recall bias are all relatively minor contributors to the total bias in this study. Furthermore, because data collectors were not the originators of the study, i.e. data collectors were unaware of the study objective; therefore, the study would have generated less sampling bias, as well as less allocation bias. Prevalence bias is likely to be the largest source of bias in this study, also referred to as Neyman bias, in which some patients with mild or severe diseases will be missing from the data collection process. In this study, the situation that emerged was the lack of patients with mild diseases. Because study data were collected from regional tertiary medical centers throughout the country, they were referred to patients with complex etiology and relatively severe diseases. Furthermore, because inclusion in the study required that patients undergo at least 1 complete etiologic screen for endocrine factors, these inclusion criteria also led to the loss of a proportion of patients with milder diseases. To address this bias, the study’s conclusions should be similarly qualified. Tertiary care centers were more likely than local primary care to use the etiologic distribution of endocrine factors for recurrent miscarriage derived from this study. Furthermore, a significant confounding factor between the number of pregnancy loss and endocrine dysfunction was age. In this study, we propose to use a stratified approach to remove the influence of confounding factors.

## Conclusion

In summary, this study describes the proportion of endocrine factor abnormalities in patients with recurrent miscarriages and finds no significant differences in endocrine factor abnormalities other than obesity between patients with recurrent miscarriages depending on the number of pregnancy losses. The findings of this study may support the screening of patients for endocrine-related aetiology after two miscarriages.

## Data availability statement

The raw data supporting the conclusions of this article will be made available by the authors, without undue reservation.

## Ethics statement

The studies involving humans were approved by the ethics committee of China Medical University. The studies were conducted in accordance with the local legislation and institutional requirements. Written informed consent for participation was not required from the participants or the participants’ legal guardians/next of kin in accordance with the national legislation and institutional requirements.

## Author contributions

LZ and YD designed the study. JZ and CL were involved in the data collection. YL and HS did the data analysis. JL advised on the conduct of the study. CQ had the conception for the study. All authors listed made important intellectual contribution to the work and approved the final version of the manuscript for publication. All authors contributed to the article and approved the submitted version.
