# Supplementary material for: Diagnostic workup of endocrine dysfunction in recurrent pregnancy loss: a cross-sectional study in Northeast China
Source: Front Endocrinol (Lausanne). 2023 Sep 18;14:1215469. doi: 10.3389/fendo.2023.1215469 (PMC10545878; doi:10.3389/fendo.2023.1215469)
Supplement: Supplementary file 1 [file Table_1.docx]

Supplementary table 1. Comparison of endocrine disorders among patients under 30 years old with different numbers of pregnancy loss

|  | Times of pregnancy loss | | | P value |
| --- | --- | --- | --- | --- |
|  | 2 | 3 | ≥4 |  |
| Endocrine dysfunction |  |  |  | 0.43 |
| No | 48(72.7%) | 12(18.2%) | 6(9.1%) |  |
| Yes | 45(67.2%) | 18(26.9%) | 4(6.0%) |  |
| PCOS |  |  |  | 0.452 |
| No | 83(70.3%) | 25(21.2%) | 10(8.5%) |  |
| Yes | 9(69.2%) | 4(30.8%) | 0(0%) |  |
| Thyroid Dysfunction |  |  |  | 0.613 |
| No | 71(68.9%) | 23(22.3%) | 9(8.7%) |  |
| Yes | 22(73.3%) | 7(23.3%) | 1(3.3%) |  |
| HPRL |  |  |  | 0.927 |
| No | 72(70.6%) | 22(21.6%) | 8(7.8%) |  |
| Yes | 16(66.7%) | 6(25.0%) | 2(6.3%) |  |
| Blood glucose abnormality |  |  |  | 0.208 |
| No | 87(71.3%) | 25(20.5%) | 10(8.2%) |  |
| IGT | 3(50.0%) | 3(50.0%) | 0(0%) |  |
| Vitamin D level |  |  |  | 0.970 |
| Normal | 4(80.0%) | 1(20.0%) | 0(0.0%) |  |
| Insufficient | 7(70.0%) | 2(20.0%) | 1(10%) |  |
| Deficiency | 82(70.7%) | 25(21.6%) | 9(7.8%) |  |
| Obesity |  |  |  | 0.750 |
| No | 85(70.8%) | 26(21.7%) | 9(7.5%) |  |
| Yes | 8(61.5%) | 4(30.8%) | 1(7.7%) |  |

Supplementary table 2. Comparison of endocrine disorders among patients aged 35 years or older with different numbers of pregnancy loss

|  | Times of pregnancy loss | | | P value |
| --- | --- | --- | --- | --- |
|  | 2 | 3 | ≥4 |  |
| Endocrine dysfunction |  |  |  | 0.542 |
| No | 23(41.8%) | 23(41.8%) | 9(16.4%) |  |
| Yes | 38(49.4%) | 25(32.5%) | 14(18.2%) |  |
| PCOS |  |  |  | 0.106 |
| No | 53(44.2%) | 47(39.2%) | 20(16.7%) |  |
| Yes | 8(66.7%) | 1(8.3%) | 3(25.0%) |  |
| Thyroid Dysfunction |  |  |  | 0.726 |
| No | 45(47.4%) | 35(36.8%) | 15(15.8%) |  |
| Yes | 16(43.2%) | 13(35.1%) | 8(21.6%) |  |
| HPRL |  |  |  | 0.690 |
| No | 45(44.6%) | 38(37.6%) | 8(17.8%) |  |
| Yes | 10(52.6%) | 7(36.8%) | 2(10.5%) |  |
| Blood glucose abnormality |  |  |  | 0.517 |
| No | 52(44.4%) | 44(37.6%) | 21(17.9%) |  |
| IGT | 5(71.4%) | 1(14.3%) | 1(14.3%) |  |
| DM | 1(100.0%) | 0(0.0%) | 0(0.0%) |  |
| Vitamin D level |  |  |  | 0.967 |
| Normal | 1(33.3%) | 1(33.3%) | 1(33.3%) |  |
| Insufficient | 13(46.4%) | 10(35.7%) | 5(17.9%) |  |
| Deficiency | 45(45.5%) | 37(37.4%) | 17(17.2%) |  |
| Obesity |  |  |  | 0.192 |
| No | 57(49.1%) | 40(34.5%) | 19(16.4%) |  |
| Yes | 4(25.0%) | 8(50.0%) | 4(25.0%) |  |

Supplementary table 3. Comparison of endocrine disorders among patients aged 30 and under 35 with different numbers of pregnancy loss

|  | Times of pregnancy loss | | | P value |
| --- | --- | --- | --- | --- |
|  | 2 | 3 | ≥4 |  |
| Endocrine dysfunction |  |  |  | 0.713 |
| No | 75(54.3%) | 53(38.4%) | 10(7.2%) |  |
| Yes | 68(50.7%) | 53(39.6%) | 13(9.7%) |  |
| PCOS |  |  |  | 0.908 |
| No | 125(52.3%) | 94(39.3%) | 20(8.4%) |  |
| Yes | 17(54.8%) | 11(35.5%) | 3(9.7%) |  |
| Thyroid Dysfunction |  |  |  | 0.935 |
| No | 109(52.4%) | 82(39.4%) | 17(8.2%) |  |
| Yes | 34(53.1%) | 24(37.5%) | 6(9.4%) |  |
| HPRL |  |  |  | 0.148 |
| No | 103(50.2%) | 83(40.5%) | 19(9.3%) |  |
| Yes | 28(66.7%) | 11(26.2%) | 3(7.1%) |  |
| Blood glucose abnormality |  |  |  | 0.057 |
| No | 133(54.5%) | 92(37.7%) | 19(7.8%) |  |
| IGT | 2(20.0%) | 5(50.0%) | 3(30.0%) |  |
| DM | 1(33.3%) | 2(66.7%) | 0(0.0%) |  |
| Vitamin D level |  |  |  | 0.889 |
| Normal | 2(40.0%) | 2(40.0%) | 1(20.0%) |  |
| Insufficient | 18(50.0%) | 15(41.7%) | 3(8.3%) |  |
| Deficiency | 121(53.8%) | 85(37.8%) | 19(8.4%) |  |
| Obesity |  |  |  | **0.017*** |
| No | 135(55.6%) | 89(36.6%) | 19(7.8%) |  |
| Yes | 8(27.6%) | 17(58.6%) | 4(13.8%) |  |
